# A biosensor based on *Coriolopsis gallica* laccase immobilized on nitrogen-doped multiwalled carbon nanotubes and graphene oxide for polyphenol detection

**DOI:** 10.1088/1468-6996/16/5/055004

**Published:** 2015-10-16

**Authors:** Sergio A Aguila, David Shimomoto, Franscisco Ipinza, Zaira I Bedolla-Valdez, José Romo-Herrera, Oscar E Contreras, Mario H Farías, Gabriel Alonso-Núñez

**Affiliations:** Centro de Nanociencias y Nanotecnología, Universidad Nacional Autónoma de México, Km. 107 carretera Tijuana-Ensenada, Ensenada, Baja California, C.P. 22860, Mexico

**Keywords:** biosensor, graphene oxide, laccase, nitrogen-doped carbon nanotube

## Abstract

The use of nanomaterials allows the design of ultrasensitive biosensors with advantages in the detection of organic molecules. Catechol and catechin are molecules that occur naturally in fruits, and their presence in products like dyes and wines affects quality standards. In this study, catechol and catechin were measured at the nanoscale by means of cyclic voltammetry. The oxidation of *Coriolopsis gallica* laccase immobilized on nitrogen-doped multiwalled carbon nanotubes (Lac/CN*_x_*-MWCNT) and on graphene oxide (Lac/GO) was used to measure the concentrations of catechol and catechin. Nitrogen-doped multiwalled carbon nanotubes (CN*_x_*-MWCNT) were synthesized by spray pyrolysis and characterized by scanning electron microscopy (SEM), transmission electron microscopy (TEM), and x-ray photoelectron spectroscopy (XPS). Covalently bonded hybrids with laccase (Lac/CN*_x_*-MWCNT and Lac/GO) were generated. Catalytic activity of free enzymes determined with syringaldazine yielded 14 584 UmL^−1^. With Lac/CN*_x_*-MWCNT at concentrations of 6.4 mmol L^−1^ activity was 9326 U mL^−1^, while enzyme activity measured with Lac/GO at concentration of 6.4 mmol L^−1^ was 9 234 U mL^−1^. The Lac/CN*_x_*-MWCNT hybrid showed higher stability than Lac/GO at different ethyl alcohol concentrations. The Lac/CN*_x_*-MWCNT hybrid can measure concentrations, not previously reported, as low as 1 × 10^−8^ mol L^−1^ by measuring the electric current responses.

## 1. Introduction

Organic molecules in solution can be detected by many different techniques. A few classical approaches are mass spectrometry, optical absorption spectroscopy and nuclear magnetic resonance (NMR). In general, these techniques require pre-treatments of samples before the analysis. Although these methods are very sensitive, they are difficult to miniaturize.

On the other hand, the detection of organic molecules by the electrochemical approach does not have this limitation. A biosensor is a device that uses specific biochemical reactions mediated by isolated enzymes, immunosystems, tissues, organelles or whole cells to detect chemical compounds usually by electrical, thermal or optical signals [1]. The signal of the biosensor may be processed by a variety of transducers, which may be electrochemical, optical, piezoelectric, calorimetric or magnetic. Advantages of the electrochemical transducer are high efficiency and ultra-low detection limits [2].

For biosensors based on direct electron transfer, redox proteins have to regenerate the electron transfer between the redox site and the electrode. Thus, substrate concentration is proportional to the current generated by the enzyme. Furthermore, the absence of a mediator is the main advantage, since no reactions interfere with the measurement [3].

A challenging area for biosensors is the correct immobilization of the enzymes on the electrode surface and the maintenance of a high and stable rate of electron transfer [3]. Laccases are a good prospect for these applications, because they are very stable and do not require a co-factor for biocatalysis [4].

Laccase belongs to the subgroup of multicopper oxidases. These enzymes have been studied in the industrial sector, such as the food, agriculture and bioremediation industries due to their high redox potential for molecules like mono-, di-, and poly-phenols [5].

To optimize electron transfer, an important factor in biosensor construction is the conductive material between the macromolecule and the electrode. Carbon nanomaterials, such as carbon nanotubes, have been used in applications such as gas sensors, biosensors, cathodes and anodes, and fuel cells, among others [6]. On the other hand, the nanomaterial graphene has been used in electronics and biological applications [7]. Both materials have the property of being conductive at room temperature.

Carbon nanotubes and graphene may be modified by oxidation with sulfuric acid to obtain carboxylate derivatives. Complex molecules such as protein receptors, enzymes, DNA and nanoparticles can then be anchored to the surface of these nanostructured materials [8].

Currently, the most common tools for measuring phenols are chromatography (liquid, gas or capillary electrophoresis), enzyme immuno-absorbent assay (ELISA) and electroanalytical techniques (polarography, cyclic voltammetry or pulse voltammetry). These methods provide reliable and accurate results at very low phenol concentrations. However, disadvantages are that measurements are performed with portable devices and have to be analyzed in a laboratory. Biosensors provide faster *in situ* measurements. Table 1S of the Supplementary Materials lists the currently used enzymes and transduction systems [9]. However, the resolution of these biosensors is not enough to detect phenolic compound trace levels.

**Table 1.** Comparison of polyphenol detection limits in recent studies.

Electrode description	Species	Detection limit (*μ*molL^−1^)	Reference
Lac/CNTs-GCE	*Coriolus versicolor*	0.66	[17]
Lac/Ap-RGOs/Chit/GCE	*Rhus vernificera*	7	[18]
Lac/MB-MCM-41/PVA	*Trametes versicolor*	0.331	[19]
Lac/Cu-OMC/Au	*Trametes versicolor*	0.67	[20]
Lac/ OMC/PVA/Au	*Trametes versicolor*	0.31	[21]
Lac-Nafion-ECNFs/GCE	n.s.	0.63	[22]
Lac/CNx-MWCNT	*Coriolopsis gallica*	0.01	This work^a^

a A graph of the concentration limit is shown in figure 5(B). n.s. not specified.

**Scheme 1.**
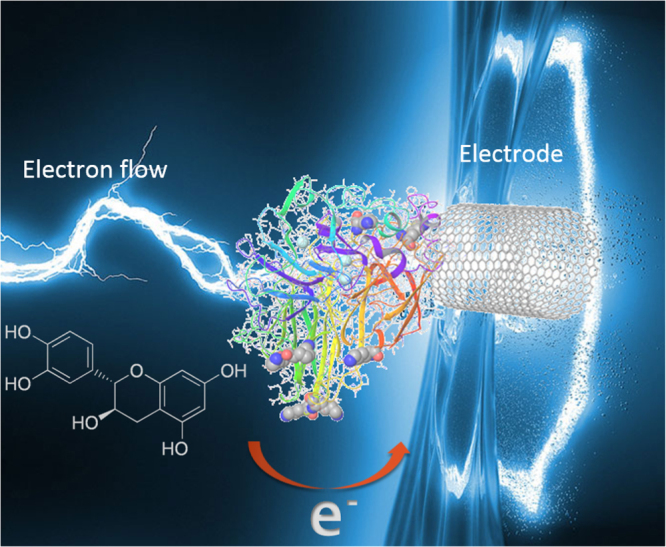
Representation of electron transfer of laccase immobilized on carbon nanotube.

In the present study, laccase immobilization on carbon nanomaterials was aimed to capture the electronic transference of catechol derivatives in the redox process of the enzyme as shown in scheme 1. The electric current was detected and measured by cyclic voltammetry. The analytes were catechol and catechin, two molecules found in dyes and wines.

## 2. Materials and methods

### 2.1. Nitrogen-doped multiwalled carbon nanotube (CN*_x_*-MWCNT) synthesis

Spray pyrolysis process has been described in previous reports [10–12].

The tubing was heated with a cylindrical furnace (Thermolyne 1200) equipped with a high precision temperature controller (±1 °C). A solution of ferrocene (98.00%) in 25 mL of toluene (98.00%) was placed in a glass container. Atomized argon (99.99%) was used as the carrier gas and it was regulated by a mass flow controller with setting at 5 L min^−1^. Once the furnace temperature reached 900 °C, an argon/benzilamine/ferrocene mixture was fed into the Vycor® tubing of 0.9 cm internal diameter and 23 cm length. The fed solution was kept constant for 15 min in all experiments. Then, the black MWCNT film formed on the inner surface of the Vycor® tube was mechanically removed with a brush and stored in concentrated nitric acid for 12 h, before washing and reducing the catalyst Fe residues.

### 2.2. SEM and TEM

The sample was morphologically characterized by scanning electron microscopy (SEM, Jeol JSM 5300) and transmission electron microscopy (TEM, Jeol JEM-2010). Specimens for TEM were prepared by casting one drop of the dispersion sample onto a standard film on copper grids. The TEM was operated at 200 kV.

### 2.3. XPS

X-ray photoelectron spectra (XPS) of sulfide catalysts were obtained in a SPECS custom-made system using a PHOIBOS 150 WAL hemispherical analyzer and a *μ*-FOCUS 500 x-ray source. All the data were obtained using monochromated Al K*α* x-rays (1486.6 eV, 110 W), a pass energy of 50 eV, and a wide angle lens mode. The diameter of the analyzed area was 0.88 mm. Under these conditions the Ag 3d^5/2^ photoelectron line was recorded with 0.630 eV full width at half maximum at a binding energy of 368.3 eV. Charge was referenced against adventitious carbon (C 1 s 284.8 eV). The analysis chamber pressure was kept under 2 × 10^−9^ mbar. Sulfide catalysts were mounted on a sample holder and kept overnight in high vacuum inside the preparation chamber before they were transferred to the spectrometer analysis chamber. The C1s, O1s, S2p and Re4f energy regions were scanned with several sweeps until a good signal-to-noise ratio was observed. Spectra are presented with and without smoothing or background subtraction.

### 2.4. Preparation of the covalent immobilization

**Figure 1.**
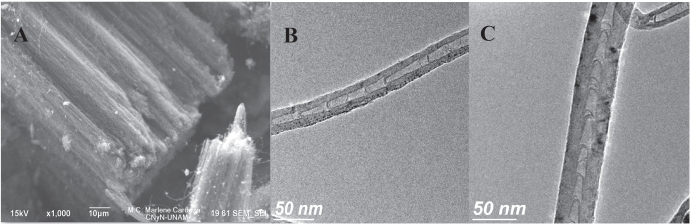
General morphology of the nitrogen-doped carbon nanotubes. (a) SEM image showing a carpet-like morphology with parallel nanotubes. (b) TEM image displaying the structure of the nanotubes, with a bamboo-like morphology typical of nitrogen-doped carbon nanotubes [13]. (c) High resolution TEM image of a nitrogen-doped carbon nanotube; notice the curvature due to the incorporation of nitrogen [13]. More figures are included in the supplementary materials (figures 2S–5S).

The carbon nanomaterials were carboxylated with nitric acid under stirring during 4 h at 80 °C. Then, they were washed with Milli-Q water and filtered using Whatman No. 1 filter paper. Thereafter, the nanomaterials were dried at room temperature and then functionalized with N-carbodiimide creating an amide bond between the amino group of the laccase (side chain of lysine) and the carboxylic groups of the nanomaterials (figure 1S). The functionalization was performed by dissolving 7.5 mg of CN*_x_*-MWCNT or 7.5 mg graphene oxide (GO), 100 *μ*L of 1-ethyl-3-(3-dimethylaminopropyl)carbodiimide (EDAC) (200 *μ*mol L^−1^), 100 *μ*L of N-hydroxysuccinimide (NHS) (200 *μ*mol L^−1^), 10 *μ*L of laccase (Lac) (44 mg mL^−1^) in 790 *μ*L of buffer phosphate. The reaction was kept at a temperature of 4 °C during 24 h with continuous agitation. Hybrids were washed three times with 1.2 mL of a phosphate buffer solution and centrifuged at 9000 rpm for 4 min. Finally, the obtained hybrids were stored in buffer phosphate solution of 100 mmol L^−1^ at pH 7.4.

### 2.5. Electrochemical experiments

Cyclic voltammetry experiments were performed with an Autolab potensiostat (PGSTAT302N) using the three-electrode configuration: Ag/AgCl [Sat. NaCl] as reference electrode, glassy carbon as working electrode (3 mm diameter) and a platinum electrode as the counter electrode. The inks of CN*_x_*-MWCNT and GO were prepared with 7.5 mg of material dispersed in 1 mL of Nafion (Sigma Aldrich, 5% in aliphatic alcohol). 2.5 *μ*L of prepared ink was placed on the glassy carbon electrode. All anode cyclic voltammetry experiments were done at room temperature. The active site of the enzyme is constituted by four copper atoms associated in three types of sites, designed as T1, T2, and T3 sites. The redox potential of the T1 site (1 Cu^2+^) is characteristic for each particular laccase and is responsible of electrons abstraction from the phenolic derivative. Then, these electrons are transferred from T1 site (1 Cu^2+^) to T2/T3 sites (3 Cu^2+^) (figure 1S).

### 2.6. Chromatography assays

To determine the activity of Lac/CN*_x_*-MWCNT and Lac/GO under different concentrations of ethanol (v:v), 10 *μ*L of the hybrids were diluted in succinate buffer, catechol (10 mmol L^−1^) and different percentages (V:V) of ethanol. The samples were measured in an a high-performance liquid chromatography (HPLC) system (Agilent) using acetonitrile as solvent and a wavelength of 260 nm with a C18 column (LiChrospher, 5 *μ*m particle size; 15 cm length).

### 2.7. Determination of enzymatic activity and of protein concentration

Laccase (Lac) activity was measured with spectrophotometer (Perkin Elmer Lambda 25), using succinate buffer and different volumes of enzyme: 10, 15, 20 and 25 *μ*L. Protein concentration was determined by the Bradford method with the BioRad protein reagent and measured at a wavelength of 595 nm.

### 2.8. Limit detection of phenolic derivatives

Assays of limit detection were carried out using logarithmic concentration of catechin since 10^−5^ to 10^−9^ mol L^−1^ in triplicate (figure 5(B)). The raw data can be observed in the table 2S (see the supplementary materials).

## 3. Results

The hybrid samples (Lac/CN*_x_*-MWCNT and Lac/GO) used as biosensors to determine catechol and catechin concentration were obtained by anchoring laccase on two types of carbon nanostructured materials: lab-synthesized nitrogen-doped multiwalled carbon nanotubes (CN*_x_*-MWCNTs) and graphene oxide (GO).

### 3.1. Electron microscopy

Figure 1 shows the general morphology of the nitrogen-doped carbon nanotubes. Figure 1(A) is a SEM image showing the carpet-like morphology of an array of parallel nanotubes. The TEM image in figure 1(B) shows the structure of the obtained nanotubes, revealing the bamboo-like configuration typical of nitrogen-doped carbon nanotubes. This has been previously observed in nanotubes synthesized in the presence of nitrogen [13]. More detail is shown in the high resolution TEM image (figure 1(C)).

### 3.2. XPS

**Figure 2.**
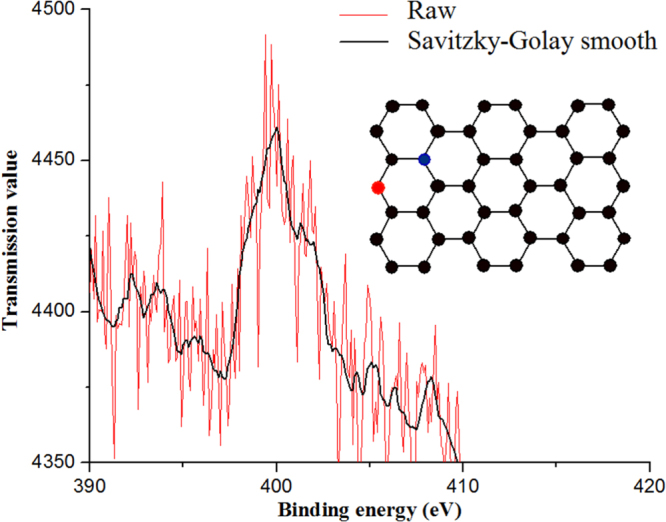
XPS of CN*_x_*-MWCNTs synthesized at 900 °C. The two main peaks are indicative of the nitrogen bonded to the nanomaterial. The red line is the raw signal and the black line corresponds to the second order Savitzky–Golay smoothing.

The XPS shown in figure 2 confirms the incorporation of approximately 5% of nitrogen with the peak centered at 400 eV, which corresponds to pyrrolic nitrogen [13]. Although the signal is weak, the peak has been deconvoluted into more components [14], quaternary nitrogen (402 eV) and pyridinic nitrogen (398.5 eV), which provide electrons to the *π* orbitals of the nitrogen atoms with pyridinic configuration [15]. Though the incorporation of nitrogen atoms is random, it is related to the type of precursor [16]. Moreover, nitrogen is the donor atom providing the metallic property of the nanotubes.

### 3.3. Activity assays and stability

In order to determine the transformation of the substrate per unit of enzyme, the catalytic activity of the free enzyme was measured using syringaldazine. The assay predicts the behavior of the enzyme under ideal conditions, and the results showed an activity of 14 584 U mL^−1^. The catalytic activity obtained with the Lac/CN*_x_*-MWCNT hybrid was 9326 U mL^−1^ and with the Lac/GO hybrid it was 9324 U mL^−1^. The lower activity obtained with the laccase hybrids may be due to diffusion effects towards the substrate when the enzyme is immobilized.

**Figure 3.**
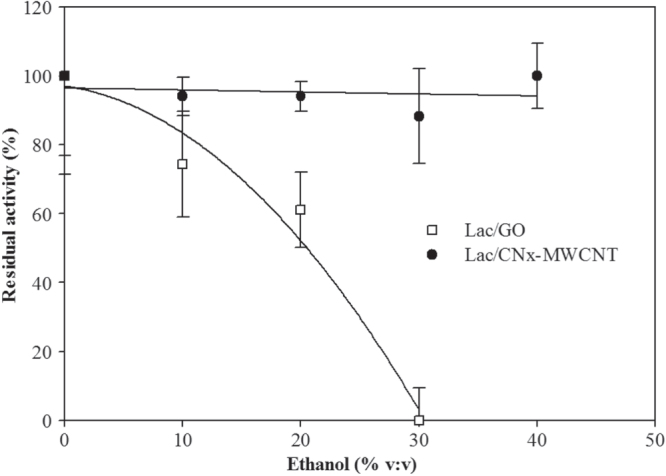
Effect of ethanol on catalytic activity using Lac/CN*_x_*-MWCNT and Lac/GO measured by HPLC.

In order to predict the operational stability of the laccase hybrids in wine or alcoholic drinks, we used high performance liquid chromatography (HPLC) with different ethyl alcohol percentages. Results showed that Lac/CN*_x_*-MWCNT remained stable, while Lac/GO stability decayed in ethanol concentrations between 10% and 25% (figure 3). The residual operational stability of Lac/CN*_x_*-MWCNT and Lac/GO was also measured and above results were confirmed (figure 6S, supplementary materials).

Using the Lineweaver–Burk equation in its electrochemical form, the Michaelis–Menten constants were calculated for catechol:

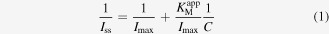
where

*I*_max_ = maximum current (A)

*I*_ss_ = stable current (A)


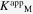
 = Michaelis–Menten constant (*μ*mol L^−1^)

*C* = substrate concentration (*μ*mol L^−1^)

For the hybrid Lac/GO, we observed a 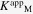 = 1.71 *μ*mol L^−1^, while for the hybrid Lac/CN*_x_*-MWCNT we obtained a dissociation constant of 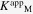 = 1.17 *μ*mol L^−1^.

### 3.4. Electrochemistry

**Figure 4.**
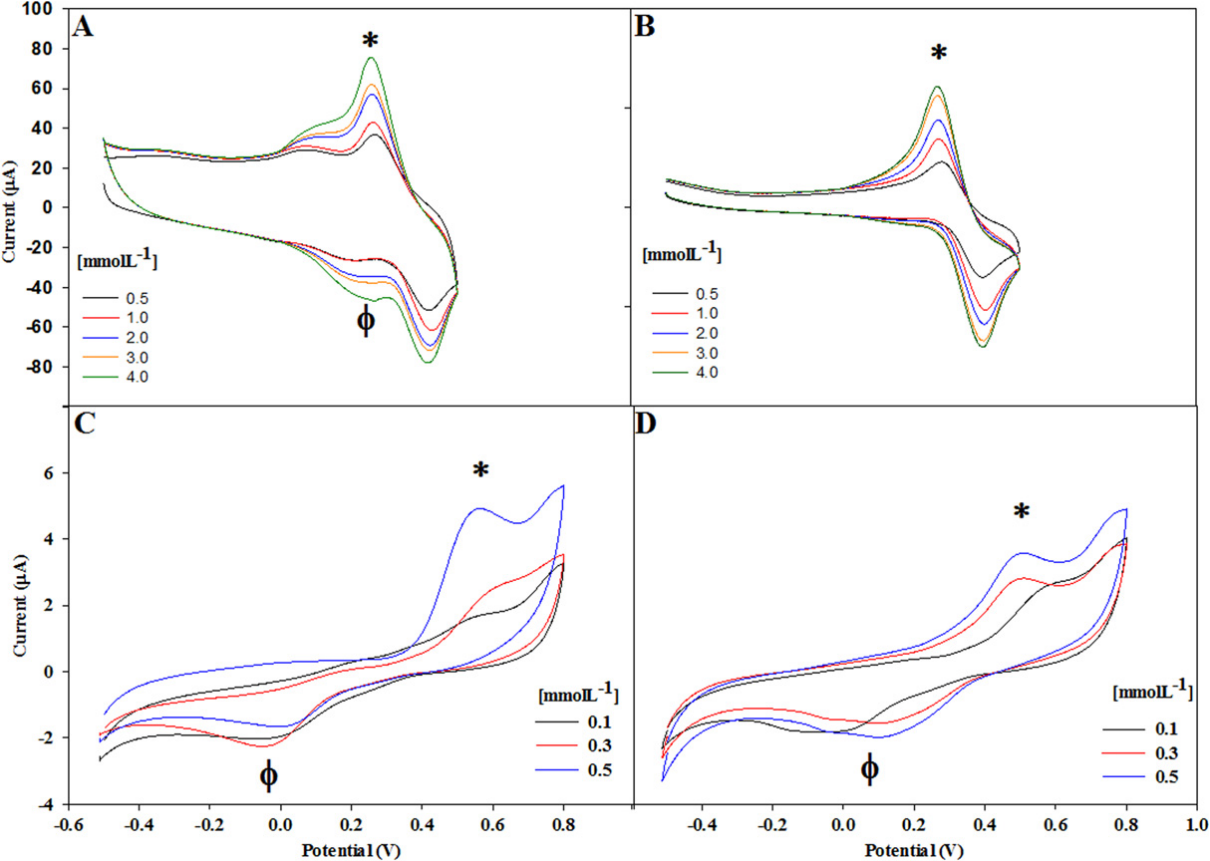
Voltammograms recorded using (A) Lac/CN*_x_*-MWCNT on catechol, (B) Lac/GO hybrid on catechol, (C) Lac/CN*_x_*-MWCNT hybrid on catechin, and (D) Lac/GO hybrid on catechin.

Electrochemical assays were performed to determine the detection level of the hybrids regarding the oxidation and reduction of mono- and poly-phenolic compounds. The voltammograms obtained using the Lac/CN*_x_*-MWCNT hybrid on catechol (figure 4(A)) showed two peaks. The first peak (∗) around 0.25 V indicates the oxidation of catechol and the second peak (*ϕ*) around 0.2 V indicates the reduction of catechol.

In figure 4(B) we show the voltammogram using the Lac/GO hybrid on catechol. The first peak (∗) around 0.25 V indicates the catechol oxidation and the second peak around 0.4 V shows the catechol reduction, i.e. a thermodynamically reversible reaction.

We show the voltammogram using the Lac/CN*_x_*MWCNT hybrid at different concentration of catechin (from 0.1 to 0.5 mmolL^−1^) in figure 4(C). The first peak (∗) around 0.55 V indicates the oxidation of catechin and the second peak (*ϕ*) around 0.0 V indicates the reduction of catechin, i.e. a thermodynamically reversible reaction. In the figure 4(D) we can observe voltammogram using the Lac/GO hybrid at different concentration of catechin (from 0.1 to 0.5 mmol L^−1^). The first peak (∗) around 0.55 V indicates the oxidation of catechin and the second peak (*ϕ*) around 0.0 V indicates the reduction of catechin, i.e. a thermodynamically reversible reaction.

A linear relationship between catechol concentration and the current generated by the oxidation of the molecule was found (figure 7S, supplementary materials).

**Figure 5.**
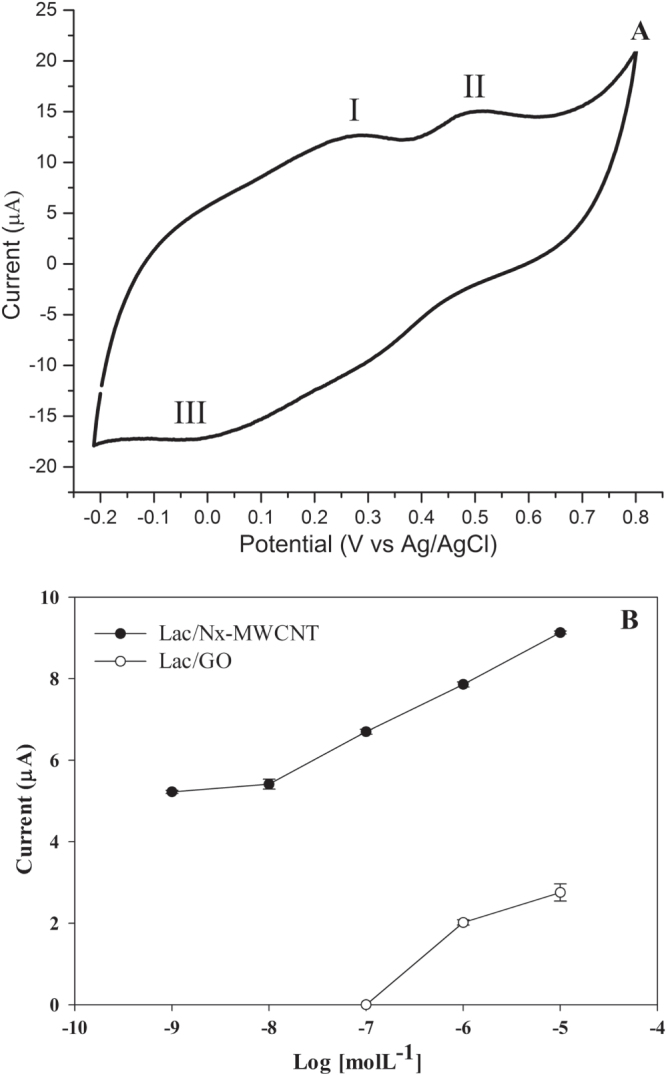
Catechin detection in white wine voltammogram. (A) Sites 1 and 2 represent the oxidation peaks and site 3 the reduction peak. This type of wine contains one of the lowest concentrations of catechin. (B) Limit of detection of catechin with laccase on CN*_x_*-MWCNT and GO. The assays were done in triplicate (table 1S, supplementary materials).

Since this biosensor will be used in the wine or fruit sector to detect catechol and catechin, an assay was devised with the more successful hybrid, Lac/CN*_x_*-MWCNT. The results showed three defined peaks, two oxidation peaks at ∼0.25 and 0.5 V and the reduction peak at ∼0.15 V (figure 5(A)).

### 3.5. Sensitivity of the hybrids and wine analysis

Laccase immobilized on CN*_x_*-MWCNT and GO were used to sense phenolic derivatives in a wine sample. The limit of detection of catechin Lac/CN*_x_*-MWCNT was at 1 × 10^−8^ mol L^−1^, while with Lac/GO it was 1 × 10^−6^ mol L^−1^ (figure 5(B)). The limit obtained with the Lac/CN_I_-MWCNT is lower than in other reports (table 1).

A Zinfandel white wine from Valle Redondo, California was analyzed using cyclic voltammetry. Figure 5(A) shows the three peaks of catechin. The peaks observed were 0.3, 0.5 and ∼0.0 V with respect to Ag/AgCl, corresponding to two oxidation peaks (I and II) and one reduction peak (III).

## 4. Discussion

Laccase was successfully immobilized onto multiwalled carbon nanotubes (MWCNT) and graphene oxide (GO) forming Lac/CN*_x_*-MWCNT and Lac/GO hybrids, and their catalytic activity to detect polyphenols in wine was confirmed.

### 4.1. Catalytic activity and stability of Lac/CN*_x_*-MWCNT and Lac/GO hybrids

In order to determine the detection efficiency for catechin, a molecule present in wine where the hybrids are intended to be used, and the effect of ethanol on the hybrids was studied. Figure 3 shows that the Lac/GO hybrid had higher catalytic activity; however, it started to decay at about 10% of ethanol concentration, while the Lac/CN*_x_*-MWCNT hybrid activity remained about constant. The effect of ethanol on proteins is due to dehydration, though at 10% this cannot generate an abrupt change in the catalytic activity because laccases are stable in wines [23]. It has been reported that ethanol is an essential solvent to form thin films [24], so when ethanol is added, the GO sheets with bonded laccase start to aggregate and an impedance is generated in the catalytic activity.

One of the most recent works using non-doped carbon nanofibers reports a *K*_M_ value 50.6 *μ*mol L^−1^ [25]. The calculated *K*_M_ constant indicates a very low dissociative enzyme–substrate relationship [26]. In contrast, laccase immobilized on CN*_x_*-MWCNT and GO showed good affinity for phenolic derivatives (1.17 and 1.71 *μ*mol L^−1^, respectively) compared with the work of Li [25].

### 4.2. Electrochemical detection of catechol derivatives

Cyclic voltammetry (CV) was used to investigate the catalytic and electrochemical behavior of both hybrids. The cathodic current in catechol was observed at 0.2 V using acetate buffer at pH 4.5 [27] and 0.25 V with succinate buffer at pH 4.5 and a reduction was observed at 0.15 V in acetate buffer and 0.18–0.2 V using succinate buffer (figures 4(A) and (B)).

The electrochemical assays using cyclic voltammetry showed a linear relationship (figure 7S) between the oxidation peaks and catechol concentration. We used two hybrids, laccase covalently bonded to MWCNT (Lac/CN*_x_*-MWCNT) and laccase covalently bonded to the edges [26] of GO (Lac/GO) at concentrations of 1 to 5 mmol L^−1^, which typically show a linear response [28]. The sensitivity of the two hybrids was very similar, and we calculated a Pearson’s *r* of 0.992 for Lac/CN*_x_*-MWCNT and of 0.990 for Lac/GO.

The study of these two hybrids showed dependence on the catalytic current. The current saturation is related to the active site of the enzyme (redox potential), up to a concentration of 1 × 10^−8^ mmol L^−1^. The Lac/CN*_x_*-MWCNT revealed a more sensitive electrochemical response, which could be related to the incorporated nitrogen and its properties. It has been calculated that the nitrogen incorporated in pyridinic mode shifts up the Fermi level of the conduction band, and this property helps to improve the conductivity of the nanotube and the transfer rate between the bioactive centers to the nanotube [15, 29]. Also, it was theoretically determined that the CN*_x_*-MWCNT show metallic behavior due to the donor states of pyridinic nitrogen [30].

Regarding bioapplications, MWCNT doped with nitrogen (CN*_x_*-MWCNT) are preferred over non-doped MWCNT. Besides the electronic properties, the structure of the CN*_x_*-MWCNT may be hydrophilic, unlike MWCNT, which are hydrophobic. The nitrogen substituted in the nanotubes generates electronic donor states. The electrons of N use three of their valence electrons to form three *σ* bonds, and two electrons fill the *π* orbitals. The N in the CN*_x_*-MWCNT facilitates electron transfer between metalloproteins and gold electrodes [31]. On the other hand, graphene is a very promising material. Basically, it is a monolayer of carbon atoms, with the same hexagonal structure of the layers forming three-dimensional graphite [32–34]. Electrons are not traversing the network impairments to carbon, so they move at constant speed (of the order of 106 m s^−1^) related to the energy of the Fermi level.

We also studied the behavior of catechin with Lac/CN*_x_*-MWCNT and Lac/GO. In these assays two peaks were present, one cathodic peak at around 0.6 V related to the resorcinol group and another cathodic peak at around 0.2 V reversible at 0.15 V, related to the catechol group [35] (figures 4(C) and (D)).

The cathodic response of the Lac/GO hybrid at around 0.6 V was adequate for 0.5 mmol L^−1^ of catechin. However, it presented a response of notoriously less sensitivity for the lower catechin concentrations of 0.3 and 0.1 mmol L^−1^. Although the oxidation and reduction peaks are large and defined, the cathodic response showed poor sensitivity to low concentrations of phenol derivatives. This response may be due to the fact that GO has low electrical conductivity, since the bond between the enzyme and the nanostructured material is essentially developed at the edges and makes the redox center not available along the entire area of the GO.

### 4.3. Wine analysis with the Lac/CN*_x_*-MWCNT hybrid and limit detection

Analysis of Zinfandel white wine from Valle Redondo, California, using cyclic voltammetry, detected three peaks corresponding to catechin (figure 5(A)). This result indicates that our Lac/CN*_x_*-MWCNT-based biosensor is a good prospect due to its metallic and redox properties, as compared to other biosensors (table 1). The incorporated nitrogen provides electron donor states that boost the electrical current generated by the oxidation of the analyte.

Nitrogen-doped MWCNT (CN*_x_*-MWCNT) were more sensitive than graphene oxide (GO) regarding alcoholic beverages, because graphene oxide folds in the presence of ethanol and the enzyme is overlapped.

The Lac/CN*_x_*-MWCNT hybrid showed more stability than Lac/GO at different ethyl alcohol concentrations and regarding the operational process. Although the catalytic activity is diminished when the enzyme is immobilized on CN*_x_*-MWCNT and GO, the detection sensitivity of phenol derivatives showed an increase in electrochemical tests. This biosensor can effectively measure polyphenol concentrations not previously reported, as low as 1 × 10^−8^ molL^−1^ (figure 5(B)) by measuring the electric current responses. In addition, the Lac/CN*_x_*-MWCNT hybrid is a promising prospect to produce a new electrode based on nanostructured materials.

## 5. Conclusions

The synthesis of nitrogen-doped multiwalled carbon nanotubes (CN*_x_*-MWCNT) by the spray pyrolysis method at a temperature of 900 °C provided high-quality doped nanotubes with 5% atomic content of nitrogen in these nanomaterials. The donor states of the pyridinic nitrogen in the nanotubes conferred more sensitive properties to CN*_x_*-MWCNT than to graphene oxide due to the metallic character, easy dispersion and stability of CN*_x_*-MWCNT in water–ethanol solutions. When the immobilized laccase in the Lac/CN*_x_*-MWCNT hybrid starts to catalyze the substrate, nitrogen incorporated into the nanotubes improves the electronic transport, which may then be detected by cyclic voltammetry. The Lac/CN*_x_*-MWCNT hybrid is a promising candidate for polyphenol detection in fruit, the wine industry and for environmental control.
